# Are Socio-Economic Indicators Associated with Food Safety in Public Schools? A Study in Sergipe State, Brazil

**DOI:** 10.3390/foods13162620

**Published:** 2024-08-21

**Authors:** Isabela Gomes Canuto, Diogo Thimoteo da Cunha, Paula Ribeiro Buarque, Izabela Maria Montezano de Carvalho

**Affiliations:** 1 Programa de Pós-Graduação em Ciências da Nutrição, Esporte e Metabolismo, Faculdade de Ciências Aplicadas, Universidade Estadual de Campinas, Unicamp, Rua Pedro Zaccaria, 1300, Caixa Postal 1068, Limeira 13484-350, São Paulo, Brazil; isabelagomesc@outlook.com; 2 Laboratório multidisciplinar em Alimentos e Saúde, Faculdade de Ciências Aplicadas, Universidade Estadual de Campinas, Unicamp, Rua Pedro Zaccaria, 1300, Caixa Postal 1068, Limeira 13484-350, São Paulo, Brazil; 3 Departamento de Nutrição, Centro de Ciências Biológicas e da Saúde, Universidade Federal de Sergipe (UFS), Av. Marechal Rondom, s/n, Jardim Rosa Elze, São Cristóvão 49100-000, Sergipe, Brazil; pbuarque@academico.ufs.br; 4 Departamento de Nutrição e Saúde, Universidade Federal de Viçosa (UFV), Av. Peter Henry Rolfs, s/n, Campus Universitário, Viçosa 36570-900, Minas Gerais, Brazil; i.montezano.c@gmail.com

**Keywords:** food safety, school feeding, geographic mapping

## Abstract

The Brazilian National School Feeding Programme aims to ensure food security and the right to food for public school students. To protect these fundamental rights, a comprehensive approach is needed that includes ensuring food safety. Recognising that low socio-economic conditions, inadequate food safety and child vulnerability can pose a cumulative burden on child development, this study examined food safety in public schools in Sergipe, Brazil, in the context of local socio-economic indicators. All state public schools in Sergipe (*n* = 314) were included. Food safety and socio-economic data were analysed using secondary sources and geographical maps. The cluster analysis identified two different groups of schools based on socio-economic indicators. While most schools presented regular foodborne illness risks, food production and temperature control had particularly high levels of non-compliance. Schools in areas with higher socio-economic indicators (Cluster 2) had better overall food safety scores (*p* < 0.001) compared to schools in areas with lower socio-economic indicators (Cluster 1). Cluster 1 schools also had a higher FBI risk when analysing temperature-controlled equipment violations (*p* = 0.001), food handlers (*p* = 0.005) and process and production (*p* = 0.004), which emerged as critical areas. These results emphasise the urgent need for targeted interventions to improve food safety in schools located in areas with lower socio-economic conditions.

## 1. Introduction

The right to adequate food is a fundamental human right, encompassing availability, accessibility, and adequacy of food for all. This indivisible right cannot be compromised by other environmental factors. In Brazil, the National School Feeding Program (NSFP—*PNAE in Portuguese*) exemplifies a significant policy initiative aimed at guaranteeing food security and ensuring this right for public school students from elementary to high school grades. Given its substantial scale and impact, the NSFP has been extensively researched in terms of its nutritional [1], environmental, food security, and food safety implications. While inextricably linked, food safety and food security represent distinct concepts. Food safety encompasses the practices and conditions necessary to prevent food contamination and illness [2], whereas food security refers to the availability, accessibility, utilisation, and stability of safe and nutritious food for all individuals [3].

Currently serving 37.9 million students nationwide, the NSFP is one of Brazil’s pioneering social assistance policies [4]. Established in 1940 and formally recognised as a right in 2009, the NSFP aims to foster the biological and psychosocial development of students, ultimately enhancing their academic performance. Aligned with guidelines for adequate and healthy meal provision, the NSFP is underpinned by principles of food and nutrition education, universal access, community engagement, and environmental sustainability [5]. All students enrolled in Brazil’s public schools have a legal entitlement to a free, adequate meal during school hours [6,7]. However, the NSFP encompasses a comprehensive approach that goes beyond mere food supply. The programme emphasises the integration of local culture and traditions while upholding stringent nutritional and health standards. A mandatory requirement for sourcing at least one third of food products from local family farms underscores the programme’s commitment to supporting agricultural communities [8]. To function optimally, the programme must navigate the complexities of food chain management and adhere to strict financial guidelines that prioritise food expenditure. However, the socio-economic context of individual school communities can influence resource allocation and impact the purchase of infrastructure and equipment [6]. Investment in physical infrastructure, such as equipment and facilities, is critical to improving or mediating food safety [9]. In addition, developing human capital through employee education, financial compensation and strong leadership is essential to fostering a positive food safety culture [10,11].

Food safety is an indispensable component of food security and encompasses all stages of food production and processing [12,13]. While it is a fundamental human right, regardless of socio-economic status, its effective realisation can be hampered by the scarcity of resources associated with socio-economic conditions. This underscores the complex interplay between food safety, food security and broader societal factors in the context of the NSFP. Research has shown a link between socio-economic factors and food safety, with low-income and minority populations having a higher susceptibility to foodborne illness (FBI). For example, research from Ethiopia has indicated a correlation between higher income, access to clean water and sanitation facilities, and improved food safety practices [14,15]. Conversely, a study from India has demonstrated that adequate hygiene practices can be maintained even in low socio-economic contexts [16]. These findings underscore the complex relationship between socio-economic factors and food safety outcomes. This is of particular concern, as children under the age of five—a population that is heavily represented in school meal programmes—are especially vulnerable to foodborne pathogens due to their still-developing immune systems. This confluence of factors underscores the complex interplay between socio-economic conditions, food safety and public health that leads to a cumulative burden on vulnerable populations.

Previous studies have documented cases of food safety deficiencies in Brazilian public schools [17] and elsewhere [18,19]. A previous study in Brazil revealed widespread non-compliance with food safety standards in public schools, including issues such as pest infestations, inadequate sanitation, and insufficient health screenings for food handlers [17]. Inadequate facilities and improper practices, such as poor hand hygiene, are commonly observed in this environment [20,21]. However, this study is the first to examine the relationship between socio-economic factors and food safety in this context. While recognising the critical role of human error and inadequate practises in FBI outbreaks [22], this study emphasises the systemic nature of the problem and highlights the need for improved resource allocation and management within school food services [9]. Food safety is an inherent component of food security, necessitating a standardised approach. There is no hierarchical distinction between different levels of food safety, as all individuals have an equal right to consume safe food. This study hypothesised a direct correlation between socio-economic indicators and FBI risk within public schools, positing that lower socio-economic areas would exhibit higher high-risk food safety violations. To investigate this potential association, the study assessed FBI risk in public schools across the state of Sergipe, Brazil.

## 2. Materials and Methods

This cross-sectional study quantitatively analysed food safety compliance and FBI risk in public schools in the Brazilian state of Sergipe. Sergipe, a state with an area of 21,915 km^2^ and over 2 million inhabitants and 75 municipalities, served as the study site. In order to analyse the relationship between food safety and socio-economic conditions, several indicators were included in the study: the Social Vulnerability Index, the Human Development Index (HDI), the Gini coefficient, the Gross Domestic Product (GDP) per capita and the Basic Education Development Index. These indices were selected because they comprehensively reflect socio-economic factors.

Secondary data from the state’s Education and Culture Department were utilised for this study. Collected in 2019 by nutritionists working within the school feeding programme, this dataset represents the most recent available data prior to the study period (2021–2023). The data encompassed 314 schools distributed across nine administrative regions, including the capital city, within Sergipe’s 75 municipalities. 

All public schools within the state, encompassing elementary and high school levels, were included in the study, representing a student population of approximately 155 thousand [23].

### 2.1. Socio-Economic Indicators

To reflect the socio-economic situation of each municipality, some indicators that may influence the hygiene practices in school food and nutrition units were listed and collected at the Brazilian Institute of Geography and Statistics’ website [24] with the exception of the Social Vulnerability Index, which was obtained through the online Social Vulnerability Atlas from the Brazilian Institute of Applied Economic Research [25].

The Social Vulnerability Index, which ranges from 0 (low) to 1 (high), was used to assess social exclusion and vulnerability in Brazilian municipalities [26]. The HDI, which ranges from 0 (low) to 1 (high), complemented this by measuring progress in income, education and health. The Gini coefficient, varying from 0 (perfect equality) to 1 (maximal inequality) was used to measure income distribution [27]. The GDP per capita, measured in Brazilian reais, was used as an indicator of general economic prosperity [28]. Finally, the Basic Education Development Index, which ranges from 0 (low performance) to 10 (high performance), was included to reflect educational outcomes consistent with the programme’s goals to improve student learning [5].

These values were gathered from the year of 2019 or the most recent ones until this year. The Gini coefficient is the least updated indicator, dating from 2003, while the HDI and Social Vulnerability Index come from the 2010 Brazilian population census, whereas the Basic Education Development Index and GDP per capita values are from 2019.

### 2.2. Food Safety Assessment

A checklist from the “Instructions Guide for Best Practises in School Feeding” [29,30] was used to assess food safety compliance. This tool quantifies FBI risk using a matrix that considers the consequences and likelihood of each violation. The checklist comprises six sections: the structure and facilities of food preparation areas (P1); controlled temperature equipment (P2); food handlers (P3); reception (P4); processes and production (P5); and environmental hygiene (P6). The answers are rated on a scale of 0 to 8 depending on the severity and importance of the risk, with a weighted value assigned to each section (k = 10, 15, 25, 30).

To determine the total section score (Px), the following formula was used:

Px = TS/(ΣTP − ΣNA) × k(1)

TS = total score; ΣTP = total of possible scores; ΣNA = total of “non-applicable” items; k = section weight.

At the end of the application, this instrument gives a general risk score (GS = P1 + P2 + P3 + P4 + P5 + P6) ranging from 0 to 100%. This score is classified in intervals of very high (0–25%), high (26–50%), regular (51–75%), low (76–90%) and very low (91–100%) sanitary risk situation [21].

### 2.3. Analysis

The data were analysed by distribution, kurtosis and deviations. Social indicators were included in a two-step cluster analysis and the absent values of Basic Education Development Index, due to shortages on the database, were permuted using linear tendency. The distance was measured by the likelihood logarithm and the cluster criteria was the Schwarz information criterion. Food safety was compared between the two well-defined clusters using Student’s t test. To normalise the variables, the bootstrap procedure was conducted using 1000 samples. To quantify the magnitude of the differences between groups, Cohen’s d effect sizes were calculated and categorised as small (d < 0.20), medium (d = 0.21–0.79), or large (d > 0.80), following Cohen’s (1988) guidelines [31]. The analyses were conducted in SPSS v.26 and JASP v.4.0.1.

To visualise spatial patterns in the data, conditional maps were generated using GeoDa software v.1.20.0.36 [32]. Variable categorization was based on box plot analysis, with quantiles used to classify socio-economic indicators.

## 3. Results

The majority of public schools in Sergipe were categorised as regular hygiene risk (n = 242), with no school falling into the lowest risk category. A total of 50 schools achieved a low-risk classification (scores between 76 and 90%), while 23 schools had a high sanitary risk. Critically, one school had an alarmingly low compliance rate of less than 25%.

Table 1 presents a regional overview of food safety scores across the state’s 75 municipalities, grouped by administrative region. Despite variations in the number of schools and municipalities per region, overall food safety compliance was relatively consistent. While a moderate level of compliance was observed across all food safety assessment blocks, the ‘Reception’ block demonstrated superior performance, as indicated by higher scores and lower standard deviations.

**Figure 1:**
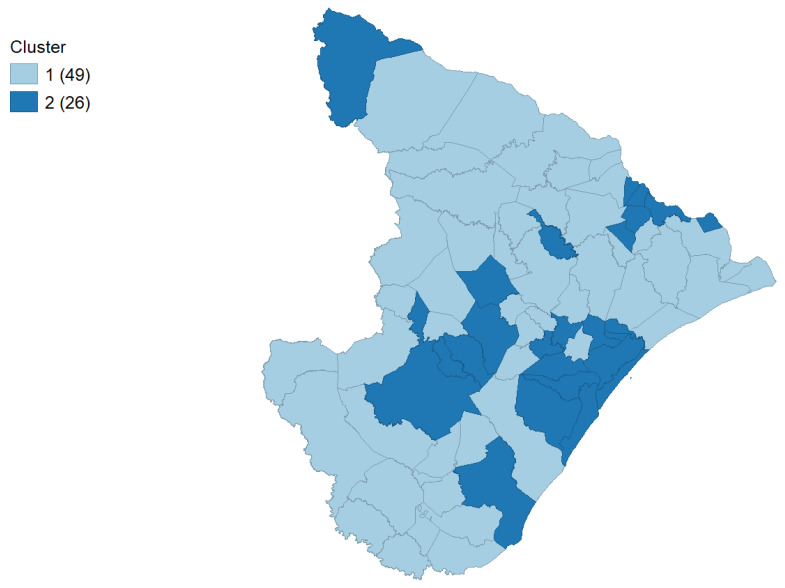
Unique values map representation of Clusters 1 (lower indicators) and 2 (higher indicators).

To further investigate food safety in this sample, the socio-economic indicators and the previous results were examined by cluster analysis, and two clearly defined clusters were found (Table 2). The clusters showed differences in HDI, GDP per capita, Gini coefficient and SVI indices, with Cluster 1 having the lower indices. In Figure 1, it is possible to see the individual clusters on the map of Sergipe, where Cluster 2 is mainly composed of the capital, Aracaju, and its surroundings.

Cluster 1 had lower scores for the P2: controlled temperature equipment, P3: food handlers, and P5: process and production, and as well as for general score (Table 3). All pairwise comparisons revealed medium effect sizes (d > 0.3), with the general score demonstrating the largest effect (d = 0.48). Interestingly, the P1: structure and facilities of food preparation areas block showed no difference between the clusters, as did the P4: reception and P5: environmental hygiene blocks. Given that the general score is a proxy for FBI risk, it can be inferred that schools within the lower socio-economic cluster (Cluster 1) are more likely to face elevated FBI risk.

**Figure 2:**
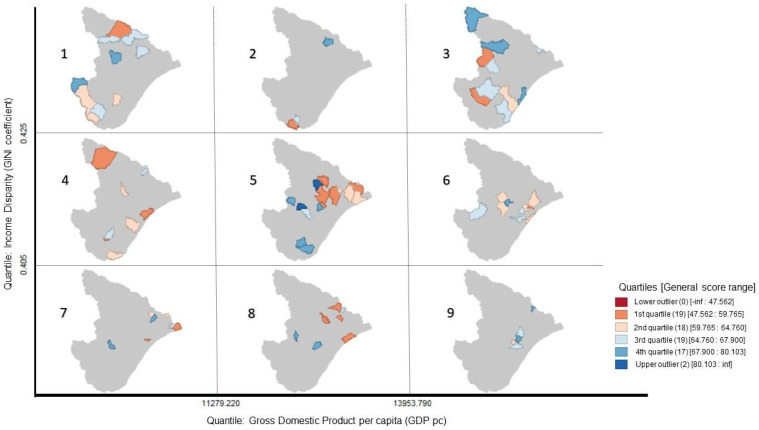
Conditional map of municipalities’ Gini coefficient, gross domestic product (GDP) per capita, and general score (GS) in the food safety assessment.

**Figure 3:**
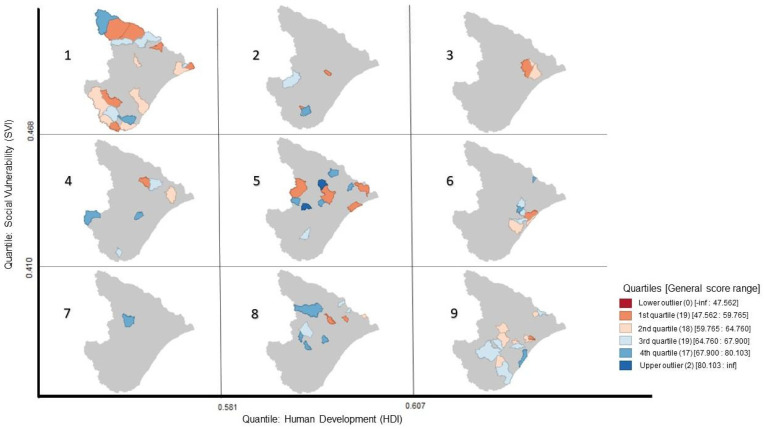
Conditional map of municipalities’ Social Vulnerability Index, Human Development Index and general score (GS) in the food safety assessment.

Considering the significance of cluster results aforementioned, conditional maps on Figure 2 and Figure 3 were elaborated to better comprehend the data of these indicators related to the food safety general score. The indicators in each map were selected by association with one another. It is valid to highlight that the classifications were based on the box plot results of the sample.

Figure 2 displays a higher accumulation of municipalities of Sergipe in the intervals from 0.405 to 0.425 of Gini coefficient and from 11,279,220 to 13,953,790 of GDP (BRL) per capita (5: Arauá, Campo do Brito, Capela, Feira Nova, Graccho Cardoso, Japoatã, Macambira, Malhador, Muribeca, Neópolis, Nossa Senhora das Dores, Pacatuba, Pinhão, Santa Luzia do Itanhy, Santa Rosa de Lima) and in both the highest indicators of the sample (3: Aracaju, Canindé de São Francisco, Carira, Estância, Frei Paulo, Itaporanga d’Ajuda, Lagarto, Nossa Senhora da Glória, Propriá, Riachão dos Dantas), showing a known reality of economic income and disparity. The lowest general scores are situated in the higher economic disparity and lower income (1: Aquidabã, Gararu, Itabaianinha, Monte Alegre de Sergipe, Nossa Senhora Aparecida, Poço Verde, Porto da Folha, Salgado, Tobias Barreto, Tomar do Geru and 4: Boquim, Indiaroba, Nossa Senhora de Lourdes, Pedrinhas, Poço Redondo, Santo Amaro das Brotas, São Cristóvão, São Miguel do Aleixo), presented by the socio-economic indicators used. This reinforces the association between these conditions and the food safety scores.

In Figure 3, there is a higher concentration of municipalities on the equivalent values of indicators, for example, the higher the SVI, the lower the HDI (1: Brejo Grande, Canhoba, Canindé de São Francisco, Cristinápolis, Gararu, Ilha das Flores, Indiaroba, Itabaianinha, Itaporanga d’Ajuda, Monte Alegre de Sergipe, Pacatuba, Poço Redondo, Porto da Folha, Riachão dos Dantas, Santa Luzia do Itanhy, São Miguel do Aleixo, Tobias Barreto, Tomar do Geru and 9: Aracaju, Campo do Brito, Carmópolis, Cedro de São João, Estância, General Maynard, Itabaiana, Lagarto, Laranjeiras, Propriá, Riachuelo, Ribeirópolis, Rosário do Catete, Salgado). The municipalities with best food safety scores are those with the lowest SVI results (7: Nossa Senhora Aparecida, 8: Cumbe, Frei Paulo, Malhada dos Bois, Malhador, Nossa Senhora da Glória, Nossa Senhora de Lourdes, Pedra Mole, Santana do São Francisco, São Domingos, Telha and 9), although there is a higher concentration on the quantiles from 0.581 to 0.607 of HDI and from 0.410 to 0.468 of Gini coefficient (5: Boquim, Carira, Feira Nova, Itabi, Macambira, Moita Bonita, Neópolis, Nossa Senhora das Dores, Pinhão, Pirambu, São Francisco). The lowest general scores are mainly situated amongst the highest level of social vulnerability.

The upper outliers and the lowest general scores belong mostly to municipalities, which have medium socio-economic values in general and do not stand out by their high or low indicators (5 and 8). All these results demonstrate the association of socio-economic indicators with the general scores of food safety practices in school environments.

## 4. Discussion

### 4.1. General Discussion

The analysis revealed significant differences between the two clusters, with schools in Cluster 1 having a higher FBI risk and poorer socio-economic conditions. These findings emphasise the challenges of the NSFP in ensuring food safety and security, particularly in socio-economically deprived areas. Given that schools are the third most common location for FBI outbreaks in Brazil, with 2019 being the second most common outbreak year between 2014 and 2023, the situation deserves urgent attention [33].

Theoretically, the highest FBI risk failures are concentrated in items from blocks P2: controlled temperature equipment, P3: food handlers, and P5: process and production, precisely the blocks with the lowest adequacy in municipalities and with the lowest socio-economic indicators. Failure to comply with food safety requirements in these specific blocks can create conditions that favour the cross-contamination, proliferation and survival of pathogenic microorganisms [34]. According to the World Health Organisation, the consumption of unsafe food leads to a cyclical pattern of disease and malnutrition that disproportionately affects vulnerable populations such as infants, children, the elderly and the sick [35].

Contrary to expectations, cities with lower socio-economic indicators did not exhibit consistently higher rates of food safety violations across all assessment blocks. While the anticipated association between socio-economic status and the condition of structure and facilities of food preparation areas was not confirmed, the observed violations in Cluster 1 may reflect underlying cultural factors influencing food safety practices, as suggested by previous research [36,37]. Effective food safety management requires a comprehensive approach that goes beyond training [10]. It requires organisational support, including the provision of adequate resources and infrastructure. Whilst training is essential, it needs to be complemented by supporting systems and practises to ensure its effectiveness. Recognising the interplay between organisational factors and food safety management systems is critical to the development of effective policies, regulations and future research [38]. Previous studies have shown the relationship between the knowledge and behaviour of food handlers and their socio-economic situation [18,39,40]. However, the present study extends these findings by examining the influence of broader systemic factors on food safety practises. The availability of adequate infrastructure, such as handwashing facilities, is critical to promoting safe food handling behaviours. This emphasises the need for a comprehensive approach to food safety that addresses both individual and systemic factors [41].

Finally, following Ferguson’s (2009) [42] criteria for a “practically” significant effect size in social sciences (d > 0.41), the observed difference in general risk scores between clusters (d = 0.48) is considered substantial and warrants the development of informed policies and recommendations. Inadequate access to sufficient, safe, and nutritious food constitutes a violation of the human right to adequate food. This study highlights the discriminatory nature of food access, as evidenced by the disparities in food safety within the NSFP across municipalities with varying socio-economic conditions in Sergipe. As advocated by Kroth et al. (2020) [43], prioritising programmes with high impact, such as the NSFP, is essential for public sector intervention. Given the intrinsic link between food safety and the right to food, a comprehensive approach is required. This involves not only strengthening regulatory frameworks but also investing in training, infrastructure, and leadership development within the school food service sector [44]. Previous research has demonstrated the effectiveness of such strategies in improving food safety outcomes [45].

### 4.2. Practical Implications

This study represents a pioneering attempt to investigate the relationship between socio-economic factors and food safety in the context of the Brazilian NSFP. The results contribute to the emerging literature on this important topic. By revealing differences in food safety in regions with different socio-economic conditions, this study emphasises the need for targeted interventions. Such interventions should prioritise the allocation of resources to schools in disadvantaged areas to improve food safety and promote equitable access to safe and nutritious food. The alignment of Brazil’s forthcoming food safety policy with the WHO’s Global Strategy for Food Safety is timely and commendable. The strategy’s comprehensive approach, which includes system strengthening, intersectoral collaboration, knowledge development, effective communication and continuous evaluation, provides a solid framework for improving food safety worldwide [46].

Therefore, to address inequities in food safety, the introduction of additional funding for schools in economically disadvantaged communities is recommended. These additional funds can be used to improve food service infrastructure and meal quality. To promote a proactive food safety culture, the implementation of robust quality management systems is paramount, prioritising schools in areas with low socio-economic indicators. While financial resources are crucial, effective leadership, organisational structure and clear communication channels are equally important to achieve improvements in food safety [47].

### 4.3. Limitations and Future Research

While this study focused on the state of Sergipe, it is acknowledged that regional variations in food safety practices exist across Brazil. Nonetheless, the findings underscore the critical need for enhanced food safety measures, particularly in regions with lower socio-economic conditions.

A limitation of this study was the reliance on slightly outdated data for the Gini coefficient, HDI, and Social Vulnerability Index. While these indicators exhibit relative stability over time, more recent data could potentially refine cluster analysis and enhance the study’s precision.

Future research should investigate the intricate relationship between food safety, food security and socio-economic factors. This includes examining how socio-economic conditions influence not only the risk of FBI, but also diet quality and food accessibility. It is important to recognise that the relationships between these indicators can be complex and nonlinear, so refined techniques are required to fully clarify their interactions.

## 5. Conclusions

The findings reveal a clear association between socio-economic conditions and food safety outcomes within schools. Municipalities categorised in Cluster 1, characterised by lower socio-economic indicators, exhibited poorer overall food safety practises and high FBI risk. Notably, controlled temperature equipment, food handlers, and process and production components emerged as critical areas of concern within these schools. These results underscore the systemic challenges in ensuring food safety in disadvantaged regions and highlight the need for targeted interventions to address these disparities.

## Figures and Tables

**Table 1:** Means and standard deviations of the results found in the assessment of food safety practices grouped by Regional Education Boards and the Aracaju Education Board in the 6 blocks.

Administrative Regions	Municipalities (n)	Schools (n)	P1 (%)	P2 (%)	P3 (%)	P4 (%)	P5 (%)	P6 (%)	GS (%)
Capital	1	78	62.9 ± 15.0	65.9 ± 27.7	77.7 ± 18.9	99.3 ± 6.2	54.3 ± 13.2	61.4 ± 16.4	67.9 ± 11.9
01	11	30	59.3 ± 12.2	58.6 ± 20.1	71.0 ± 27.6	96.3 ± 19.3	50.4 ± 11.1	63.5 ± 15.4	63.7 ± 10.5
02	5	38	63.0 ± 11.1	55.8 ± 20.5	70.4 ± 22.2	99.5 ± 3.0	51.9 ± 10.6	66.1 ± 17.5	64.4 ± 8.4
03	14	34	60.7 ± 13.2	65.3 ± 26.0	77.4 ± 19.3	96.3 ± 12.4	51.4 ± 9.3	58.5 ± 12.2	65.9 ± 8.7
04	9	16	59.4 ± 10.7	51.9 ± 23.4	62.6 ± 24.8	97.0 ± 11.7	45.4 ± 8.7	54.3 ± 17.8	58.1 ± 8.7
05	6	10	61.8 ± 15.0	49.8 ± 25.2	71.7 ± 20.5	100.0 ± 0.0	52.8 ± 16.2	59.0 ± 13.9	63.3 ± 11.7
06	14	35	59.3 ± 12.1	46.9 ± 23.8	74.7 ± 16.6	97.3 ± 17.2	48.8 ± 10.8	60.2 ± 21.3	62.0 ± 9.8
07	3	13	59.5 ± 9.0	48.4 ± 25.6	72.7 ± 24.6	100.0 ± 0.0	50.0 ± 9.5	49.0 ± 11.8	61.3 ± 8.6
08	8	56	61.4 ± 9.3	54.8 ± 25.7	72.7 ± 15.4	98.0 ± 14.3	51.9 ± 11.6	63.4 ± 16.2	64.2 ± 8.9
09	4	14	65.6 ± 7.8	55.1 ± 17.8	68.9 ± 27.8	100.0 ± 0.0	49.5 ± 13.0	64.1 ± 13.5	63.3 ± 9.8

Legend: P1: Structure and facilities of food preparation areas, P2: controlled temperature equipment, P3: food handlers, P4: reception, P5: processes and production, P6: environmental hygiene; GS: general score.

**Table 2:** Cluster analysis applied on the municipalities’ socio-economic indicators: Human Development Index (HDI), Basic Education Development Index, Gross Domestic Product (GDP) per capita, Gini coefficient and Social Vulnerability Index.

Socio-Economic Indicators	Cluster 1 (n = 49)	Cluster 2 (n = 26)	*p*-Value
HDI	0.578	0.629	**<0.001**
Basic Education Development Index	3.35	3.45	0.35
GDP per capita	12,788.37	19,598.15	**0.005**
Gini coefficient	0.421	0.406	**<0.001**
Social Vulnerability Index	0.466	0.389	**<0.001**

Bold values are significant differences *p* < 0.05.

**Table 3:** Comparison of food safety assessment scores by cluster.

Food Safety Assessment Variables	Cluster 1 (n = 135) Mean ± SD	Cluster 2 (n = 179) Mean ± SD	*p*-Value *	Effect-Size (d)
P1: Structure and facilities of food preparation areas	60.3 ± 11.3	62.4 ± 12.8	0.13	0.17
P2: Controlled temperature equipment	55.5 ± 22.3	63.6 ± 25.7	**0.001**	**0.33**
P3: Food handlers	71.5 ± 22.3	78.9 ± 19.0	**0.005**	**0.36**
P4: Reception	99.3 ± 12.6	97.7 ± 10.2	0.93	0.13
P5: Process and production	50.3 ± 11.5	54.6 ± 11.7	**0.004**	**0.37**
P6: Environmental hygiene	61.8 ± 16.2	61.1 ± 9.6	0.727	0.04
General risk score	63.4 ± 9.6	68.2 ± 10.3	**<0.001**	**0.48**

* Student *t*-test; legend: standard deviation (SD); bold values are significant differences *p* < 0.05.

## Data Availability

The original contributions presented in the study are included in the article, further inquiries can be directed to the corresponding author.
